# Hypertrophic Cardiomyopathy-Associated CRYAB^R123W^ Activates Calcineurin, Reduces Calcium Sequestration, and Alters the CRYAB Interactome and the Proteomic Response to Pathological Hypertrophy

**DOI:** 10.3390/ijms26062383

**Published:** 2025-03-07

**Authors:** Andres Thorkelsson, Chun Chou, Audrey Tripp, Samia A. Ali, Jonas Galper, Michael T. Chin

**Affiliations:** 1 MD Program, Tufts University School of Medicine, Boston, MA 02111, USA; andres.thorkelsson@tufts.edu (A.T.); chouch@pennmedicine.upenn.edu (C.C.); 2 Molecular Cardiology Research Institute, Tufts Medical Center, Boston, MA 02111, USA; aetripp@pm.me (A.T.); jonas.galper@tuftsmedicine.org (J.G.); 3 Genetics, Molecular and Cellular Biology Program, Tufts Graduate School of Biomedical Sciences, Boston, MA 02111, USA; samiaaali@outlook.com

**Keywords:** hypertrophic cardiomyopathy, cardiac hypertrophy, CRYAB, alpha-B-crystallin, molecular chaperone, calcineurin, transverse aortic constriction, mass spectrometry, AlphaFold

## Abstract

Hypertrophic cardiomyopathy (HCM) is the most common inherited cardiovascular condition in the world, affecting around 1 in 500 people. HCM is characterized by ventricular wall thickening, decreased ventricular chamber volume, and diastolic dysfunction. Inherited HCM is most commonly caused by sarcomere gene mutations; however, approximately 50% of patients do not present with a known mutation, highlighting the need for further research into additional pathological mutations. The alpha-B crystallin (CRYAB) mutation CRYAB^R123W^ was previously identified as a novel sarcomere-independent mutation causing HCM associated with pathological NFAT signaling in the setting of pressure overload. We generated stable H9C2 cell lines expressing FLAG-tagged wild-type and mutant CRYAB, which demonstrated that CRYAB^R123W^ increases calcineurin activity. Using AlphaFold to predict structural and interaction changes, we generated a model where CRYAB^R123W^ uniquely binds to the autoinhibitory domain of calcineurin. Co-immunoprecipitation using the CRYAB FLAG tag followed by mass spectrometry showed novel and distinct changes in the protein interaction patterns of CRYAB^R123W^. Finally, mouse heart extracts from our wild-type CRYAB and CRYAB^R123W^ models with and without pressure overload caused by transverse aortic constriction (TAC) were used in global proteomic and phosphoproteomic mass spectrometry analysis, which showed dysregulation in cytoskeletal, metabolomic, cardiac, and immune function. Our data illustrate how CRYAB^R123W^ drives calcineurin activation and exhibits distinct changes in protein interaction and cellular pathways during the development of HCM and pathological cardiac hypertrophy.

## 1. Introduction

Hypertrophic cardiomyopathy is a pathological condition defined by the thickening of the ventricular wall, decreased ventricular chamber volume, and diastolic dysfunction in the absence of predisposing factors and is the most common inherited cardiovascular condition affecting around 1 in 500 individuals worldwide [1]. It is inherited in an autosomal dominant pattern with a penetrance of around 50–60% [2,3]. Linkage analyses of large families with HCM have identified mutations in multiple cardiac sarcomere genes, including β-myosin heavy chain (*MYH7* OMIM# 160760), myosin binding protein C3 (*MYBPC3* OMIM# 600958), and cardiac troponin T (*TNNT2* OMIM# 191045) [4,5,6,7,8,9]. Although the most common mutations involved in HCM are in the cardiac sarcomere genes, they only account for around 30% of mutations found in patients, while a staggering 40% of patients do not carry any known HCM mutation [10,11]. Therefore, identifying and studying additional elusive causative genes outside the sarcomere is essential to fully understand the pathogenic mechanisms behind hypertrophic cardiomyopathy [12].

Mutant CRYAB was first implicated in cardiomyopathy decades ago in a genetic linkage study by a group of French researchers [13]. CRYAB is a member of the small heat shock protein family, which are molecular chaperones that are involved in a wide range of cellular functions, including the regulation of calcium signaling [14], protein folding and aggregation [15,16,17], autophagy [18,19], and apoptosis [20,21]. Under normal physiological conditions, CRYAB forms dimers and oligomers, which mask the N- and C-terminal unstructured regions of CRYAB and thereby inhibit its function [22,23]. Following cellular stress, CRYAB de-oligomerizes, exposing its N- and C-terminal domains, which leads to phosphorylation at different serine residues and its activation as a molecular chaperone [24,25]. CRYAB oligomers are stabilized by ionic bridges at aspartic acid residue 109 (D109) and arginine residue 120 (R120) between interacting CRYAB proteins [22,23]. Proper maintenance of CRYAB oligomers is vital for the normal functioning of the protein and prevention of aberrant protein interactions as mutations at D109 and R120 have been implicated in various familial cardiomyopathies [26]. Several mutations have been noted at D109, but the most well studied is *CRYAB*^D109G^, which causes restrictive cardiomyopathy secondary to abnormal desmin aggregation [27]. Similarly, disruptive mutations at R120 have been implicated in desmin-related cardiomyopathy, with the most common mutation being *CRYAB*^R120G^ [28]. CRYAB^R120G^ acts in a dominant negative manner, disrupting the normal functioning of wild-type CRYAB, and promotes protein aggregation, as was noted in cryoelectron microscopy of CRYAB^R120G^ mutant cells with quaternary CRYAB^R120G^ structures that were double the size of those in wild-type CRYAB cells [29,30]. A recently discovered novel mutation, *CRYAB*^R123W^, was found to cause concordant development of HCM in monozygotic twins, suggesting its pathogenic nature [31]. Mouse studies of *Cryab*^R123W^ have demonstrated a spontaneous development of diastolic dysfunction with aging and pathological hypertrophy with systolic dysfunction secondary to TAC-induced pressure overload [32]. CRYAB has also been shown to bind to calcineurin in co-immunoprecipitation experiments, and CRYAB^R123W^ has been implicated in pathological calcium signaling [32]. Abnormal CRYAB structure secondary to the R123W mutation has been shown to have an increased B-sheet content and large structural changes adjacent to its mini-chaperone peptide sequence, which is implicated in reduced chaperone activity, while simultaneously increasing the propensity of the mutant protein to form aggregates [33].

In this study, we demonstrate that CRYAB^R123W^ leads to increased calcineurin activity in H9C2 cells and impaired calcium uptake in mutant mouse cardiomyocytes. Structural analysis of CRYAB interaction with calcineurin using AlphaFold v2.2.0 suggests that CRYAB^R123W^ binds to the autoinhibitory domain of calcineurin, supporting a possible mechanism of autoinhibition release for the increased calcineurin activity. Co-immunoprecipitation and global proteomic and phosphoproteomic mass spectrometry analysis revealed differential protein binding and regulation secondary to CRYAB^R123W^, indicating distinctive cellular changes compared to other studied CRYAB mutations.

## 2. Results

### 2.1. CRYAB^R123W^ Increases Calcineurin Activity

**Figure 1:**
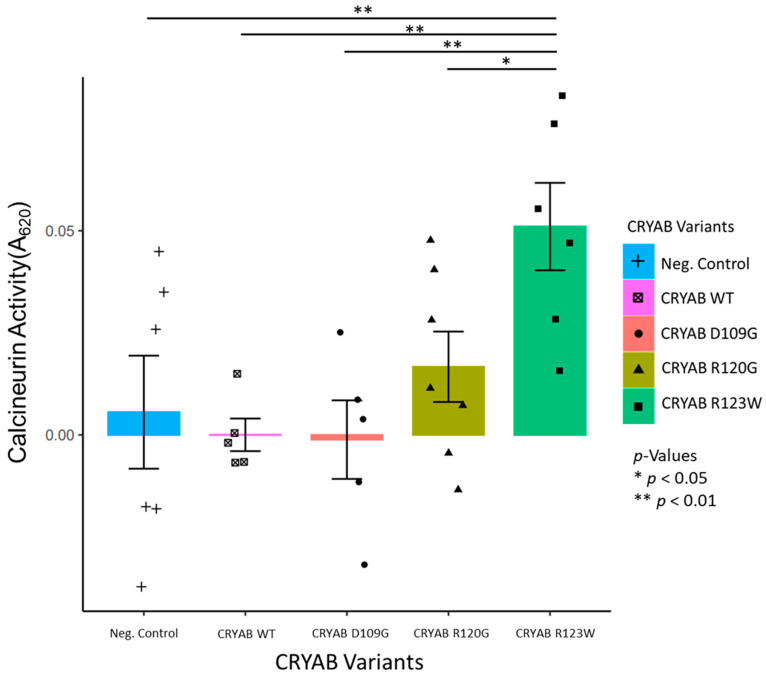
Calcineurin activity based on the calculated absorbance, representing the phosphate released by calcineurin for negative control (blue) H9C2 cells and H9C2 cells expressing FLAG-tagged wild-type (pink), D109G (red), R120G (yellow), and R123W CRYAB (green). CRYAB^R123W^ shows a significant increase in calcineurin activity compared to the other CRYAB variants.

To investigate the impact that CRYAB protein mutations have on calcineurin activity, we generated stable H9C2 cell lines overexpressing FLAG-tagged wild-type CRYAB, CRYAB^D109G^, CRYAB^R120G^, and CRYAB^R123W^ via lentivirus transduction. We measured calcineurin activity directly in cell lysates generated from these cell lines and an H9C2 negative control expressing normal levels of wild-type CRYAB using a colorimetric calcineurin activity assay. We found a significant increase in calcineurin activity in cells overexpressing CRYAB^R123W^ compared to those expressing the other two mutant proteins or wild-type protein (Figure 1). The overexpression of CRYAB, CRYAB^D109G^, or CRYAB^R120G^ did not promote an increase in calcineurin activity when compared to the negative control. These results indicate that the R123W variant of CRYAB is uniquely able to activate calcineurin when compared to the wild type or the D109G or R120G variants.

### 2.2. CRYAB^R123W^ Causes Impaired Calcium Reuptake and Myocyte Relaxation

**Figure 2:**
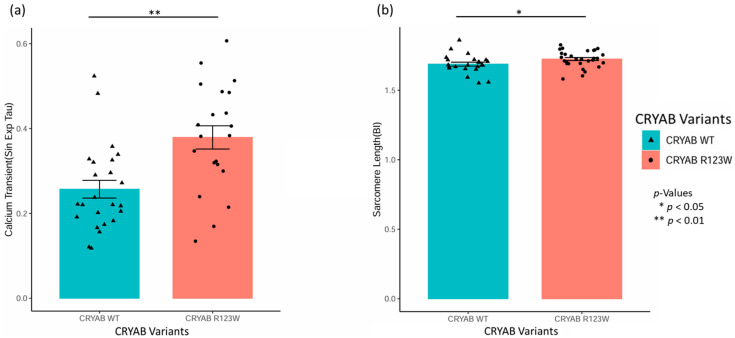
Bar plots of IonOptix analysis of isolated cardiomyocytes from our wild-type CRYAB (blue) and CRYAB^R123W^ (red) mouse models. (**a**) IonOptix Sin Exp Tau, a measure of calcium reuptake, for wild-type CRYAB and CRYAB^R123W^ is shown, with a significantly increased value in CRYAB^R123W^, which suggests impaired calcium reuptake and myocyte relaxation. (**b**) IonOptix baseline sarcomere length, demonstrating that CRYAB^R123W^ myocytes have significantly increased baseline sarcomere length compared to the wild type. * *p* < 0.05, ** *p* < 0.01.

To examine changes in cardiomyocyte contraction and calcium handling, we isolated wild-type CRYAB and CRYAB^R123W^ myocyte cells from mouse hearts and then performed an IonOptix-based assessment and analysis. No significant differences were seen in the fractional shortening. We found that compared to wild-type myocytes, CRYAB^R123W^ myocytes had significantly impaired calcium reuptake and myocyte relaxation compared to wild-type CRYAB, as measured by the elevation in single exponential tau (Figure 2a). Interestingly, we also saw that, overall, CRYAB^R123W^ myocytes have a small but significant increase in sarcomere length compared to their wild-type counterparts (Figure 2b). This provides evidence that the CRYAB R123W promotes dysregulation of calcium handling in mouse myocytes, which is consistent with the activation of calcineurin activity, NFAT reporter activity, and diastolic dysfunction, as shown in this study and reported previously [32].

### 2.3. AlphaFold Predicts That CRYAB^R123W^ Binds to the Autoinhibitory Domain of Calcineurin

**Figure 3:**
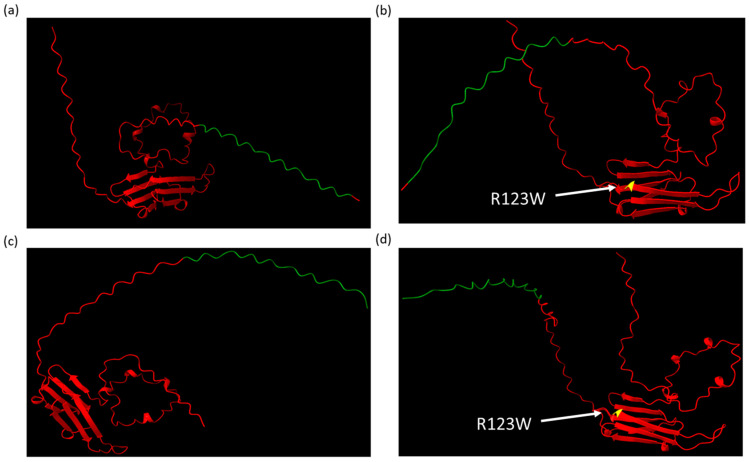
AlphaFold structures visualized in ChimeraX of CRYAB proteins (red) tagged with a triple FLAG tag (green) and mutated residues (yellow with white label): (**a**) wild-type CRYAB with N-terminal FLAG tag; (**b**) CRYAB^R123W^ with N-terminal FLAG tag; (**c**) wild-type CRYAB with C-terminal FLAG tag; and (**d**) CRYAB^R123W^ with C-terminal FLAG tag.

**Figure 4:**
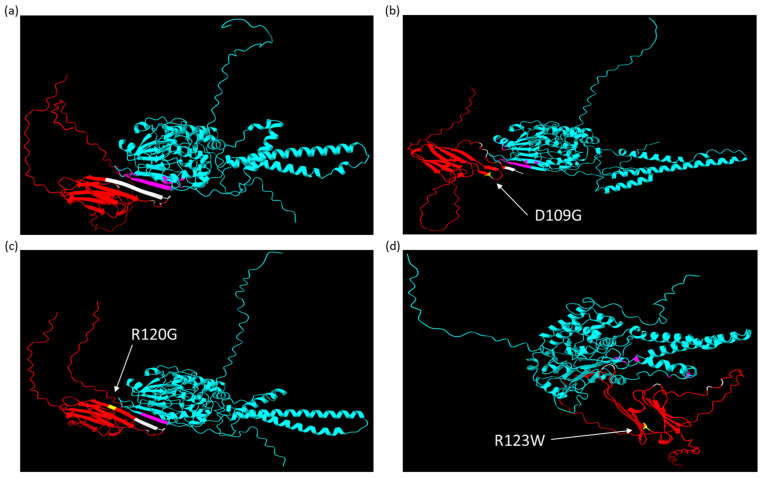
AlphaFold structures visualized in ChimeraX of CRYAB proteins (red) interacting with calcineurin (blue), with labeling of the predicted interacting amino acids of CRYAB (white) and calcineurin (pink) and the mutated CRYAB residue (yellow with white label): (**a**) wild-type CRYAB binding well at the NFAT binding site of calcineurin; (**b**) CRYAB^D109G^ binding poorly at the NFAT binding site of calcineurin; (**c**) CRYAB^R120G^ binding poorly at the NFAT binding site of calcineurin; and (**d**) CRYAB^R123W^ binding well at the autoinhibitory domain of calcineurin.

To attempt to gain a better understanding of the protein structure of the CRYAB variants and their possible interaction with calcineurin, we used the AlphaFold v2.2.0 structural prediction software [34,35]. The sequences for calcineurin and wild-type CRYAB were collected from the AlphaFold Protein database and altered with the selected mutations [36,37]. First, we wanted to ensure that the addition of a triple FLAG tag to the structure was not predicted to have significant alterations in protein structure. A triple FLAG tag sequence was added at the N- and C-terminals of the wild type and CRYAB^R123W^ mutant sequences in AlphaFold v2.2.0, which were then visualized and compared in ChimeraX v1.9 [38], with minimal alterations in the protein structure following FLAG tag addition (Figure 3a–d). Second, we modeled the interaction of wild-type CRYAB, CRYAB^D109G^, CRYAB^R120G^, and CRYAB^R123W^ with calcineurin (Figure 4a–d). As has been previously published, wild-type CRYAB binds at the NFAT binding site of calcineurin, blocking NFAT from binding to calcineurin [39] (Figure 4a). Both CRYAB^D109G^ and CRYAB^R120G^ are predicted to interact with the NFAT binding site but to a lesser extent compared to the wild type (Figure 4b,c), consistent with a loss of competition for the NFAT binding model for promoting NFAT activation and cardiac hypertrophy. CRYAB^R123W^, unlike the other proteins, was not predicted to bind at the NFAT binding site and thus would have complete loss of competition for NFAT binding; it was also uniquely predicted to bind at the autoinhibitory domain of calcineurin (Figure 4d). This suggests that unlike the other variants, CRYAB^R123W^ might have both a loss of competition for NFAT binding to calcineurin and an additional activating function by relieving the intrinsic autoinhibitory function of calcineurin to promote NFAT activation and cardiac hypertrophy. This offers a likely explanation for the significant increase in calcineurin activity seen in CRYAB^R123W^ compared to the other mutants.

### 2.4. CRYAB^R123W^ Protein Interactome Diverges from CRYAB and CRYAB^R120G^

**Figure 5:**
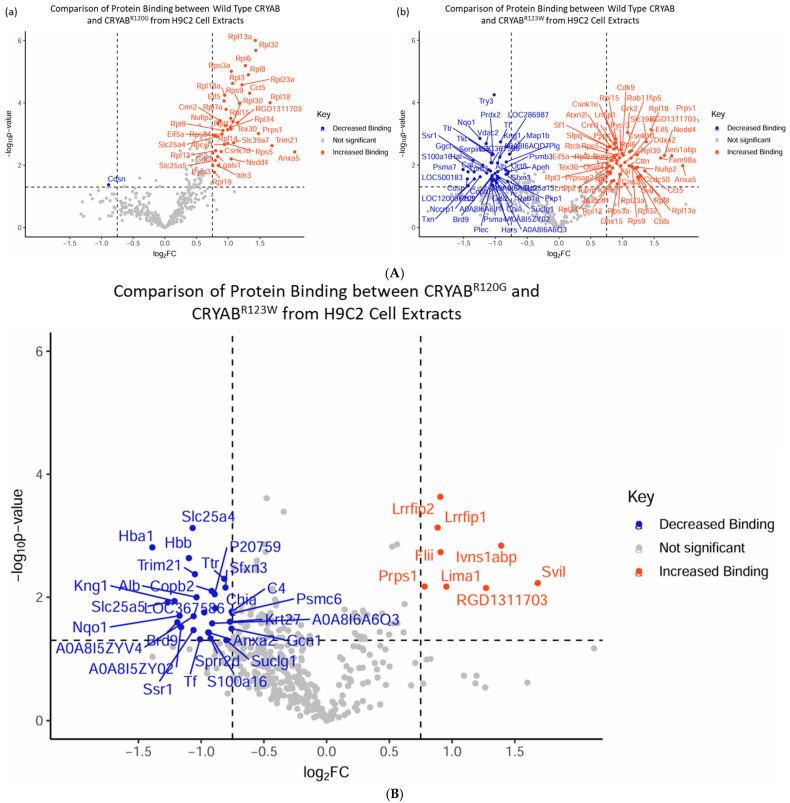
(**A**) Volcano plots depicting mass spectrometry data from co-immunoprecipitation of lysate from H9C2 cells expressing FLAG-tagged wild-type CRYAB, CRYAB^R120G^, and CRYAB^R123W^. Data are labeled with the corresponding protein and as having decreased binding (blue), no significant change (grey), or increased binding (red). (**a**) Volcano plot comparing wild-type CRYAB to CRYAB^R120G^, showing significant increase in binding with minimal decrease. (**b**) Volcano plot comparing wild-type CRYAB to CRYAB^R123W^, showing significant decreases and increases in protein binding, comparatively. (**B**) Volcano plot depicting mass spectrometry data from co-immunoprecipitation of lysate from H9C2 cells expressing FLAG-tagged CRYAB^R120G^ and CRYAB^R123W^. Data are labeled with the corresponding protein and as having decreased binding (blue), no significant change (grey), or increased binding (red). The comparison between CRYAB^R120G^ and CRYAB^R123W^ shows that CRYAB^R123W^ has distinct changes in protein–protein interactions. For all analyses, significance was based on a protein having both a *p*-value less than 0.05 and fold change greater than 2 graphed as horizontal −log_10_(*p*-value) and vertical log_2_(FC) dotted lines respectively.

To examine the impact that CRYAB mutations have on cellular protein interactions, we analyzed FLAG antibody-based co-immunoprecipitation data from H9C2 cellular extracts of FLAG-tagged wild-type CRYAB compared to FLAG-tagged CRYAB^R120G^ and CRYAB^R123W^ with unbiased LC-MS proteomics. We found that despite their relative closeness in structure and mutation location, CRYAB^R120G^ and CRYAB^R123W^ mutations have distinct patterns of changes in protein binding. When compared to wild-type CRYAB, CRYAB^R120G^ seems to mostly have increased binding to proteins, consistent with its documented propensity to form protein aggregates (Figure 5A(a)) [29,30]. While CRYAB^R123W^ has both increased and decreased binding to proteins, which is consistent with a documented loss of chaperone activity, it has increased aggregation (Figure 5A(b)) [33]. When comparing CRYAB^R120G^ and CRYAB^R123W^, we saw that the changes in protein binding between the two CRYAB mutants were distinct (Figure 5B). CRYAB^R123W^ seems to have higher binding with cytoskeletal proteins, particularly proteins involved in actin binding, such as Svil [40] and Lima1/EPLIN [41] (Figure 5B). However, CRYAB^R123W^ seems to have decreased binding to proteins involved in transcription, including Brd9 [42] and Gcn1 [43], protein degradation, such as Trim21 [44] and Nqo1 [45], and the calcium binding ion channel Anxa2 [46] (Figure 5B). Differential protein binding seen with CRYAB^R123W^ could be a driving force behind cellular pathway changes involved in the development of the HCM in contrast to the DCM phenotype seen with CRYAB^R120G^ or the restrictive CM phenotype seen with CRYAB^D109G^.

### 2.5. Global Proteomics and Phosphoproteomics Reveal Cytoskeletal, Metabolic, Immune, and Cardiac Dysfunction in CRYAB^R123W^ Mouse Hearts

**Figure 6:**
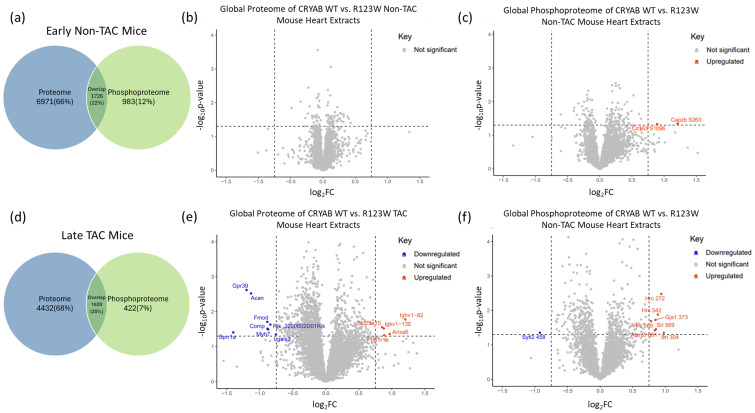
Mass spectrometry data from wild-type CRYAB and CRYAB^R123W^ mouse heart extracts without (Early Non-TAC) or with (Late TAC) transverse aortic constriction. (**a**) Venn diagram depicting the protein (blue) and phosphoprotein (green) breakdown in mice without transverse aortic constriction. (**b**) Volcano plot showing a lack of significant changes in protein expression in mice without transverse aortic constriction. (**c**) Volcano plot showing only two significant changes in phosphoprotein expression in mice without transverse aortic constriction. (**d**) Venn diagram depicting protein (blue) and phosphoprotein (green) breakdown in mice with transverse aortic constriction. (**e**) Volcano plot showing significant changes in protein expression in mice with transverse aortic constriction. (**f**) Volcano plot showing significant changes in phosphoprotein expression in mice with transverse aortic constriction. For all analyses, significance was based on a protein having both a *p*-value less than 0.05 and fold change greater than 2 graphed as horizontal −log_10_(*p*-value) and vertical log_2_(FC) dotted lines respectively.

To investigate the changes in cellular protein composition and pathways, we compared wild-type CRYAB and CRYAB^R123W^ global mouse heart extracts with and without pressure overload in the form of TAC. For the mice without TAC, we found 6971 unique proteins, 983 unique phosphorylated proteins, and 1726 proteins common to both (Figure 6a). For mice with TAC, we found 4432 unique proteins, 422 unique phosphorylated proteins, and 1620 proteins common to both (Figure 6d). Without additional pressure overload, the *CRYAB^R123W^* mutation does not induce an HCM phenotype in our mouse model [32], a common condition of HCM mouse models [47] as few develop spontaneous HCM [48,49,50,51]. Unsurprisingly, without TAC, we observed few significant changes in protein expression or phosphorylation (Figure 6b,c). However, following TAC and the emergence of a pathological hypertrophic phenotype, we observed a significant change in expression of proteins and phosphorylated proteins (Figure 6e,f), notably a downregulation of Grp39, Lgals3, and Sytl3(P), while Anax6, Ppp1r1b, Slc25a15, Hrc(P), and Atxn2l(P) were upregulated (Figure 6e,f). Given that the HCM phenotype does not develop unless there is additional stress on the heart, we designated the pathology of mouse hearts that have not undergone TAC as models of early-stage disease (Figure 6a–c) and those with TAC as models of late-stage disease (Figure 6d–f). As previously described by our lab group, the CRYAB^R123W^ mouse model developed systolic dysfunction following TAC [32], a poor prognostic factor of patients with HCM [52,53], illustrating how our mouse model mimics late and severe stages of HCM pathology.

**Figure 7:**
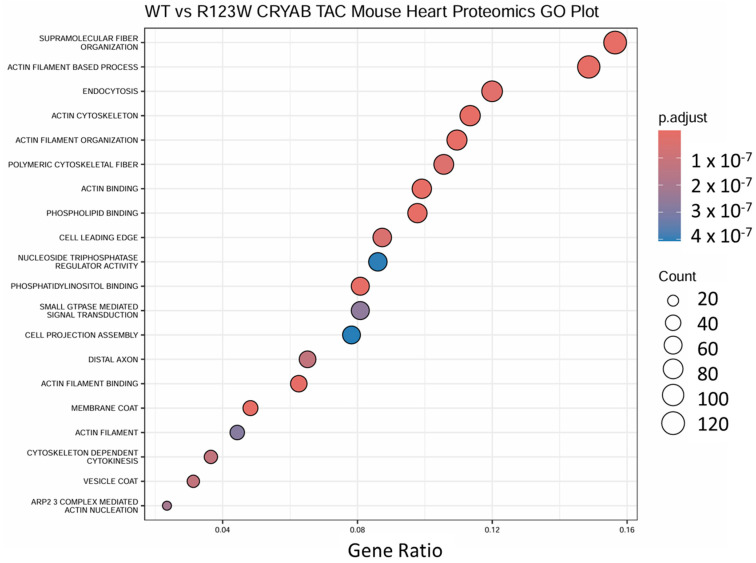
GO term analysis comparing mass spectrometry data from wild-type CRYAB and CRYAB^R123W^ mouse heart extracts following transverse aortic constriction. GO term analysis showed significant association with cytoskeletal pathways.

**Figure 8:**
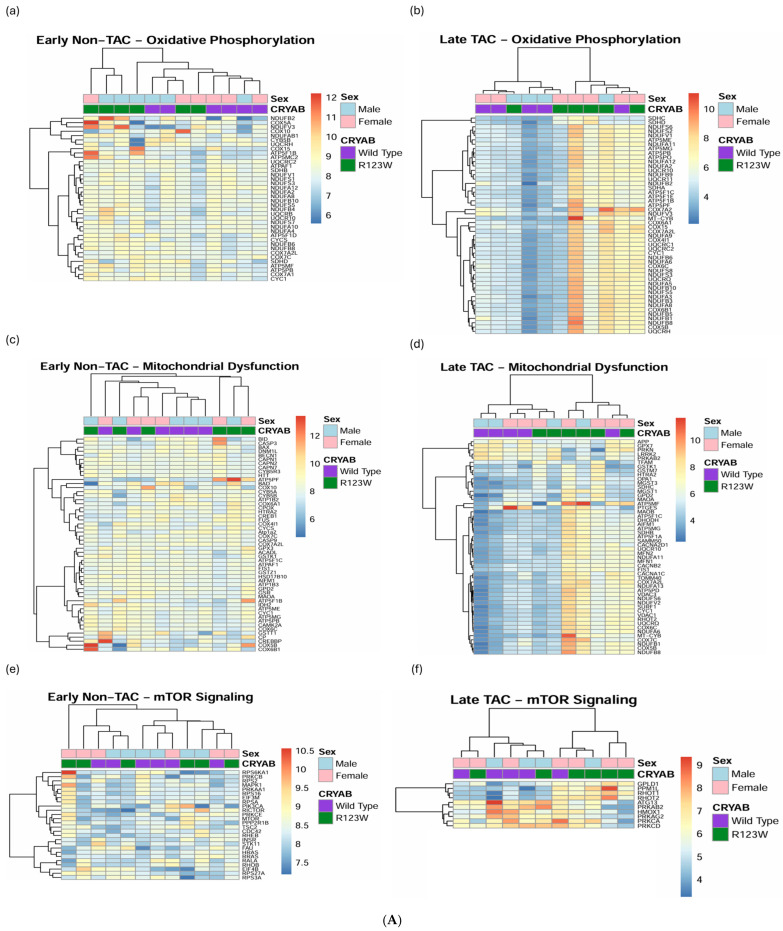

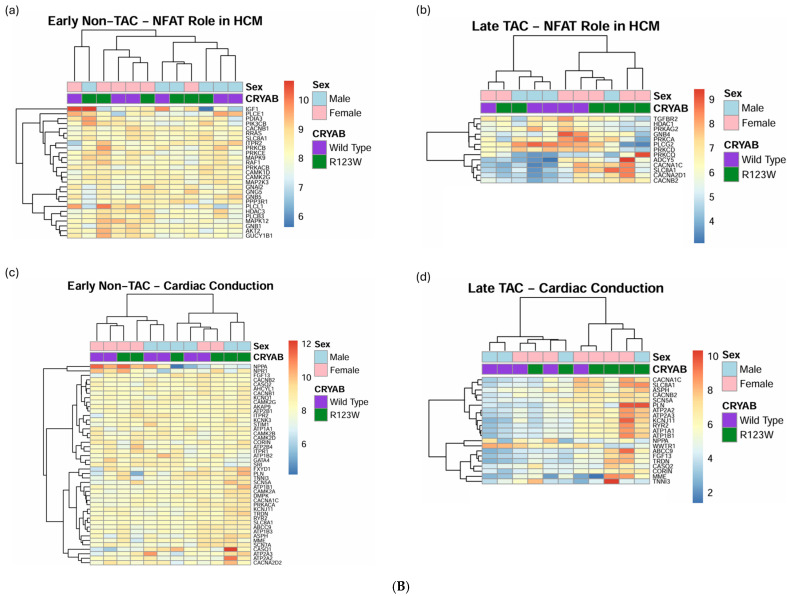

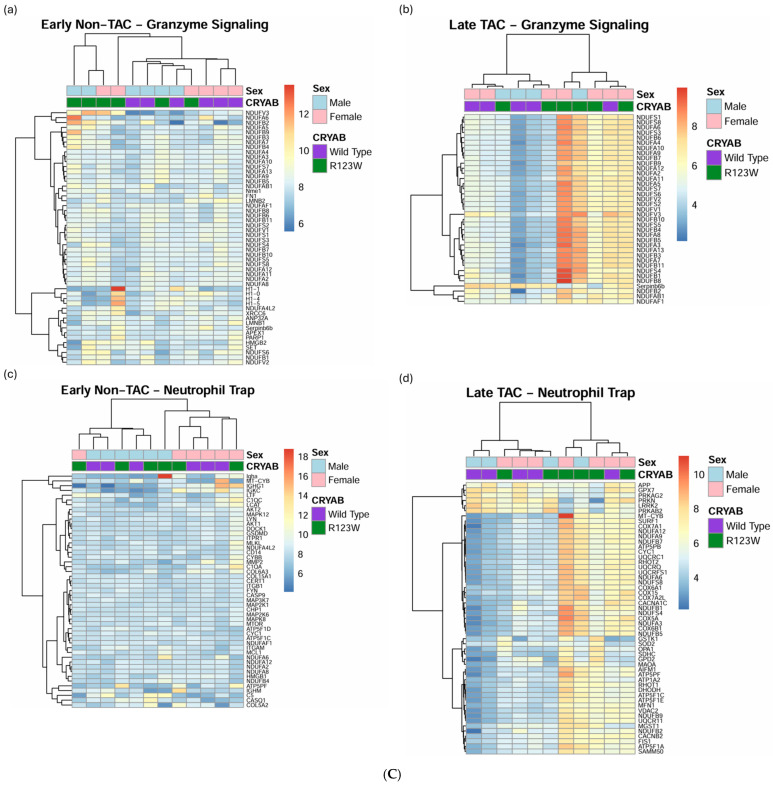
(**A**) Heatmaps based on genes from oxidative phosphorylation (**a**,**b**), mitochondrial dysfunction (**c**,**d**), and mTOR signaling (**e**,**f**) pathways identified by IPA using mass spectrometry data from wild-type CRYAB (purple) and CRYAB^R123W^ (green) mouse heart extracts with or without transverse aortic constriction. (**a**,**c**,**e**) Heatmaps showing results from early-stage disease in mice without transverse aortic constriction with poor clustering and gene expression differentiation. (**b**,**d**,**f**) Heatmaps showing results from late-stage disease in mice with transverse aortic constriction with improved clustering and gene expression differentiation. (**B**) Heatmaps based on genes from the role of NFAT in hypertrophic cardiomyopathy (**a**,**b**) and cardiac conduction (**c**,**d**) pathways identified by IPA using mass spectrometry data from wild-type CRYAB (purple) and CRYAB^R123W^ (green) mouse heart extracts with or without transverse aortic constriction. (**a**,**c**) Heatmaps showing results from early-stage disease in mice without transverse aortic constriction with poor clustering and gene expression differentiation. (**b**,**d**) Heatmaps showing results from late-stage disease in mice with transverse aortic constriction with improved clustering and gene expression differentiation. (**C**) Heatmaps based on genes from granzyme signaling (**a**,**b**) and neutrophil trap (**c**,**d**) pathways identified by IPA using mass spectrometry data from wild-type CRYAB (purple) and CRYAB^R123W^ (green) mouse heart extracts with or without transaortic constriction. (**a**,**c**) Heatmaps showing results from early-stage disease in mice without transverse aortic constriction with poor clustering and gene expression differentiation. (**b**,**d**) Heatmaps showing results from late-stage disease in mice with transverse aortic constriction with improved clustering and gene expression differentiation.

Pathway analysis of the mass spectrometry proteins using GO term analysis in R [54] showed significant associations with cytoskeletal structure and actin filament pathways (Figure 7). However, to determine if there are other possible pathways involved, we analyzed the mass spectrometry data using QIAGEN Ingenuity Pathway Analysis v127006219 (QIAGEN Inc., Hilden, Germany, https://digitalinsights.qiagen.com/IPA, accessed on 1 November 2023) (IPA) [55] software to compare the early-stage-disease and late-stage-disease mouse hearts via heatmaps. IPA revealed significant changes in pathways involved in metabolism, cardiovascular function, and immune regulation, with more congruent clustering using heatmap analysis of the global mass spectrometry data. The most significant pathways that showed increased clustering when comparing early and late stages of the condition included oxidative phosphorylation (Figure 8A(a,b)), mitochondrial dysfunction (Figure 8A(c,d)), and mTOR signaling (Figure 8A(e,f)) for metabolic pathways, NFAT involvement in HCM (Figure 8B(a,b)) and cardiac conduction (Figure 8B(c,d)) for cardiovascular pathways, and granzyme signaling (Figure 8C(a,b)) and neutrophil trapping (Figure 8C(c,d)) for immune pathways. Finally, we used IPA to analyze upstream regulators predicted to be involved in the changes seen in the protein expression of the late-stage disease mass spectrometry data, which showed significant involvement of enzymes, kinases, G protein-coupled receptors (GPCR), phosphatases, and ion channels (Table 1). Our analysis demonstrates how the stress of pressure overload reveals pathological hypertrophy pathways secondary to CRYAB^R123W^ mutation. Specifically, there are distinct changes secondary to CRYAB^R123W^-induced pathological hypertrophy in the cytoskeletal, metabolic, cardiac, and immune pathways. Finally, distinct upstream regulators are predicted to be involved in the changes seen with pathological hypertrophy secondary to CRYAB^R123W^ following pressure overload by TAC, which could be targets for future therapeutic intervention.

## 3. Discussion

In this study, we used both cell lines and mouse models to examine the cellular effects of mutations in the CRYAB protein. For our comparative mutations, we chose *CRYAB*^D109G^ and *CRYAB*^R120G^ as they are the most extensively studied *CRYAB* mutations causing cardiomyopathy, allowing us to perform an in-depth comparative analysis. In addition to being the most studied, both of these mutations are located within the same critical B-sheet domain as CRYAB^R123W^; therefore, we were interested in understanding their functional difference and pathophysiology. Previous studies have demonstrated that wild-type CRYAB is a protein interactor of calcineurin and suppresses the activity of calcineurin in the setting of pressure overload, but they did not explain how heterozygosity for CRYAB^R123W^ conferred susceptibility to pathological hypertrophy [32]. In a simple competition for the NFAT binding model, wild-type CRYAB present in CRYAB^R123W^ heterozygotes would still inhibit the interaction of NFAT with calcineurin and therefore limit calcineurin dephosphorylation of NFAT and the activation of hypertrophic signaling. Our results show that calcineurin activity was significantly elevated in cells overexpressing mutant CRYAB^R123W^, despite the continued presence of wild-type CRYAB, indicating a unique link between the mutation and aberrant calcineurin activity, which is not observed with CRYAB^D109G^ or CRYAB^R120G^. Our structural prediction using AlphaFold v2.2.0 indicates a possible interaction between CRYAB^R123W^ and the autoinhibitory domain of calcineurin, a key intrinsic regulatory motif [56]. This predicted interaction likely provides the mechanism behind the observed significant and unique increase in calcineurin activity. Our structural prediction, however, has limitations, such as the inability to model the effect of post-translation modifications and assess several known phosphorylation sites of CRYAB [24,25]. Our analysis also does not model the interaction between CRYAB and calcineurin using different constraints on the interaction, such as specific interacting configurations or amino acids, which could further illustrate the nature of their interaction.

Hypertrophic cardiomyopathy has been characterized by dysregulated fibrosis, immune regulation, inflammation, and metabolism [57]. Our global proteomics analysis demonstrates that HCM secondary to the expression of mutant CRYAB^R123W^ leads to dysregulated oxidative phosphorylation and mitochondrial function pathways. Additionally, hearts from mice carrying the CRYAB^R123W^ mutation exhibit a significant dysregulation of immune function and cytoskeletal pathways that are likely secondary to the impaired chaperone activity of CRYAB^R123W^ [33]. Wild-type CRYAB is significantly expressed in cardiac tissue [58] and has been shown to readily bind to various protein interactors [59,60], including proinflammatory proteins [61], thus having an anti-inflammatory effect [62,63,64]. Therefore, it is not surprising that mutant CRYAB proteins are involved in dysregulated inflammatory and immune responses. The role of immune and inflammatory dysregulation on the pathogenesis of HCM secondary to CRYAB^R123W^ raises the question of whether there is a role for anti-inflammatory therapeutics in the treatment of HCM.

HCM pathology has been characterized by enhanced calcium signaling regardless of the underlying pathological mutation or cause [65,66]. Wild-type CRYAB plays a crucial role in the regulation of pathological calcium signaling in the setting of pressure overload through the modulation of NFAT and calcineurin activity [32,67]. We observed a significantly impaired calcium reuptake in CRYAB^R123W^ mutant myocytes, indicating that the mutant has a role in the dysregulation of cellular calcium handling and an altered calcineurin interaction. Further investigation into the role that calcineurin and calcium play in the development of HCM in general and in the setting of CRYAB^R123W^, as well as the viability of calcineurin inhibitors as a therapeutic intervention for HCM patients, is required. Our work supports the notion that precision intracellular targeting could be crucial for the development of a treatment for HCM patients.

CRYAB has been well documented as a molecular chaperone in a variety of different tissues across the body. Previously documented mutations in CRYAB have been linked to the development of various cardiomyopathies via the disruption of ionic bridges critically involved in the molecular chaperone function of CRYAB [22,23]. CRYAB^R123W^, however, is unique in its development of HCM [31,32]. Our comparative analysis of CRYAB^R120G^ and CRYAB^R123W^ co-immunoprecipitation data suggests a distinct mechanism behind the cellular changes seen with CRYAB^R123W^; further research could illuminate novel mechanisms for the development of HCM. Recent structural studies on CRYAB^R123W^ demonstrated reduced chaperone activity and increased aggregation to cytoskeletal proteins [33]. Similarly, our co-immunoprecipitation experiments showed significant overall loss of protein binding with CRYAB^R123W^ as well as increased binding to cytoskeletal proteins and greater involvement in cytoskeletal pathways. Of note, our Co-IP cell models did not show specific binding changes to desmin for CRYAB^R123W^, which is consistent with previous publications regarding the protein [32]. Additionally, we did not see desmin binding with both the wild-type CRYAB and CRYAB^R120G^ cell models, which might be because the interaction with desmin is not stable during immunoprecipitation. Further validation of changes in interaction partners secondary to the CRYAB^R123W^ mutation could illuminate possible targets for therapeutic intervention. As a molecular chaperone, CRYAB is responsible for preventing aberrant protein folding and interactions; as such, it is likely the abnormal function of the binding partners of CRYAB that lead to the development of HCM. The identification of key CRYAB interactors affected by mutations in the protein could be targets for small molecular therapies that might also be applicable to non-CRYAB based HCM.

## 4. Materials and Methods

AlphaFold Protein Structure Modeling—Native amino acid sequences for CRYAB and calcineurin were downloaded from their respective AlphaFold Protein Structure Database entries [36,37]. CRYAB sequences were edited into their mutated form by replacing the corresponding amino acids: D109G, R120G, and R123W. All sequences (individual and multiple) were run in AlphaFold v2.2.0 using the multimer setting [34,35]. Structures with the highest fidelity, which were predicted using AlphaFold v2.2.0 ranked analysis, were considered. Images of structures were viewed and labeled using ChimeraX v1.9 developed by UCSF [38].

CRYAB DNA Insert and Plasmid Preparation—The plasmid backbone was based on the pLJM1-Empty plasmid construct (Addgene, Watertown, MA, USA; Addgene plasmid #91980; http://n2t.net/addgene:91980, accessed on 1 September 2023; RRID:Addgene_91980) [68]. CRYAB gene inserts were created using IDT gene block fragment technology (IDT Inc., Coralville IA, USA; https://www.idtdna.com/page, accessed on 1 September 2023). Gene insert sequences were created with a 40 BP Gibson homology sequence from the pLJM1 plasmid at the 5′ and 3′ ends and then internally flanked by restriction enzyme sites for NheI at the 5′ end and EcoRI at the 3′ end. Between the 5′ and 3′ ends, the main sequences consisted of a Kozak site, start codon, triple FLAG sequence, CRYAB or CRYAB sequences, triple FLAG sequence for both N- and C-terminal labeling, and a stop codon. Wild-type, D109G, R120G, and R123W CRYAB DNA sequences were used for the inserts and were retrieved from Uniprot (https://www.uniprot.org/, accessed on 1 September 2023). Linearized pLJM1-Empty plasmid was prepared using FastDigest Green Buffer (ThermoFisher Scientific, Waltham, MA, USA; ThermoFisher #B72) and NheI (ThermoFisher Scientific, Waltham, MA, USA; ThermoFisher #ER0971) and EcoRI (ThermoFisher Scientific, Waltham, MA, USA; ThermoFisher #ER0271) restriction enzymes. The solution was then incubated at 37 °C for 30 min and then at 80 °C for 5 min to inactivate the restriction enzymes. CRYAB gene inserts were incorporated into the linearized pLJM1 plasmid using Gibson assembly according to the manufacturer’s protocol (ThermoFisher Scientific, Waltham, MA, USA; ThermoFisher #A46633) [69].

Bacterial Transformation—Stable competent *E. coli* cells (New England Biolabs, Inc., Ipswich, MA, USA; NEB #C3040) were transformed using the heat shock method, as previously described, using 100 ng of the Gibson reaction solution [70]. Transformed *E. coli* cells were then grown on LB plates with ampicillin selection for 12 h before individual colonies were selected and cultured in LB media with ampicillin selection prior to plasmid isolation.

Plasmid Isolation and Sequencing—Stable *E. coli* cultures were pelleted, and plasmids were isolated using QIAGEN miniprep (QIAGEN Inc., Hilden, Germany, QIAGEN #27104) or Takarabio Midiprep (Takara # 740422.50) according to the manufacturer’s protocol. Prior to further usage, plasmids were sequenced using Sanger sequencing to confirm proper insert sequences at the Tufts University Sequencing Core.

Cell Lines—H9C2 cells purchased from ATCC (American Type Culture Collection, Manassas, VA, USA; CRL-1446) were maintained on DMEM media supplemented with 10% fetal bovine serum. H9C2 cell lines expressing triple FLAG-tagged wild-type human CRYAB, D109G, R120G, or R123W variants were generated using DNA sequences encoding the variants that were inserted to a pLenti-puro vector (Addgene, Watertown, MA, USA; #39481, Addgene). H9c2 cells were transduced with 3rd generation CRYAB-expressing lentivirus, packaged as previously described [71]. Transduced cells were selected and maintained on 10 µg/mL puromycin.

Stable CRYAB H9C2 Cell Line Preparation—Using autoclaved forceps and sterile filter paper dipped in trypsin, individual colonies of transfected H9C2 cells were selected and grown in DMEM media supplemented with 10% fetal bovine serum and puromycin to make stable cell lines. Stable H9C2 CRYAB cell lines were checked for CRYAB expression using Western blotting with a primary monoclonal mouse anti-FLAG antibody (MerckMillipore, Dramstadt, Germany; MerckMillipore #F1804) and secondary immunofluorescent AlexaFluor488 goat anti-mouse light chain-specific antibody (Jackson ImmunoResearch, West Grove, PA, USA; Jackson ImmunoResearch #115-545-174). Wild-type and mutant CRYAB cell lines with approximately equal expression were selected for use in future experiments and maintained in DMEM media supplemented with 10% fetal bovine serum and puromycin.

Calcineurin Activity Assay—CRYAB wild-type and mutant H9C2 stable cells were grown to 75% confluence on 10 cm plates before being harvested and lysed with 1 mL of ice-cold lysis buffer (50 mM HEPES buffer, 150 mM NaCl, 0.1% NP-40, Halt protease, and phosphatase inhibitors (ThermoFisher Scientific, Waltham, MA, USA; ThermoFisher # 78442)). Plates with lysis buffer were left on ice for 5 min before cells were detached with a cell scraper and collected in an Eppendorf tube. Cells were centrifuged at 12,000× *g* for 10 min at 4 °C, and then, the lysate supernatant was collected. Protein concentration was determined by BCA assay, and equal protein amounts were used between samples (approximately 1 mg/mL concentration). Calcineurin activity was measured using a colorimetric calcineurin assay kit (MerckMillipore, Dramstadt, Germany; MerckMillipore #207007), with each sample being prepared in triplicate and as specified by the manufacturer. Samples were read at 620 nm absorbance, and calcineurin activity was calculated based on the following equation: (Total − Background) − (EGTA − Background).

Co-immunoprecipitation (Co-IP)—CRYAB wild-type and mutant H9C2 stable cells were grown to 75% confluence on 10 cm plates before being harvested and lysed with 1 mL of ice-cold lysis buffer (50 mM HEPES buffer, 150 mM NaCl, 0.1% NP-40, Halt protease, and phosphatase inhibitors (ThermoFisher Scientific, Waltham, MA, USA; ThermoFisher # 78442)). Plates with lysis buffer were left on ice for 5 min before cells were detached with a cell scraper and collected in an Eppendorf tube. Cells were centrifuged at 12,000× *g* for 10 min at 4 °C, and then, the lysate supernatant was collected. Bradford assays were performed on all lysate samples, and 1 mg of each lysate was incubated with 2 µg monoclonal mouse anti-FLAG antibody (MerckMillipore, Dramstadt, Germany; MerckMillipore #F1804) and incubated on a rotator at 4 °C for 2 h. Then, 30 µL of 50% Protein G Sepharose 4 Fast Flow beads (MerckMillipore, Dramstadt, Germany; MerckMillipore #GE17-0618) were added to the lysate and rotated at 4 °C for an additional 45 min. After incubation, the tubes were centrifuged at 10,000× *g* for 30 s at 4 °C and the supernatant was removed. The remaining beads were washed with 200 µL of cold PBS and centrifuged at 10,000× *g* for 30 s 3 times. Co-immunoprecipitation samples were eluted off the beads using a 5% Formic acid solution and then centrifuged at 12,000× *g* for 30 s. The supernatant was collected and stored in Eppendorf tubes. Co-immunoprecipitation samples were lyophilized prior to shipment to the Harvard Mass Spectrometry Core for preparation as per their protocols, described below.

CRYAB Mouse Model Procedures and Global Proteomics Sample Preparation—CRYAB mouse models for both wild-type CRYAB and CRYAB^R123W^ were based on the model previously established in our lab [32]. All mice underwent echocardiography once between 10 and 12 weeks of age and then 5 weeks later as per the protocol previously described [32]. Then, 10–12-week-old wild-type CRYAB and CRYAB^R123W^ mice were respectively split into two groups, one without intervention and one to undergo severe (27G needle) transverse aortic constriction to induce pressure overload, as previously described [72]. Following the procedure, the mice were allowed to recover for 5 weeks. After the second echocardiography, all mice were anesthetized with isoflurane before being euthanized with cervical dislocation. The chest was cleaned with alcohol before being cut open, and the heart was harvested by transecting the great vessels just above their exit from the heart. The hearts were washed and chambers were emptied using ice-cold PBS before being placed in an Eppendorf tube and flash-frozen in liquid nitrogen. The hearts were stored at −80 °C until all the requisite hearts were ready for mass spectrometry preparation. Prior to mass spectrometry analysis, the hearts were homogenized in a lysis buffer (50 mM HEPES buffer, 150 mM NaCl, 0.1% NP-40, Halt protease, and phosphatase inhibitors (ThermoFisher Scientific, Waltham, MA, USA; ThermoFisher # 78442)) using an ultrasonic cell disrupter and centrifuged; then, the lysate supernatant was collected. Heart lysate samples were lyophilized prior to shipment to the Harvard Mass Spectrometry Core for preparation as per their protocols, described below. All mice were handled in accordance with the US National Institutes of Health standards, and all procedures were approved by the Tufts University Institutional Animal Care and Use Committee.

Co-IP Mass Spectrometry Sample Preparation—Dried IP elutions were solubilized in a buffer of 200 mM EPPS (pH 8.5), 8M urea, and protease inhibitors. A buffer exchange was carried out using a modified SP3 protocol [73]. Briefly, ~250 µg of Cytiva SpeedBead Magnetic Carboxylate Modified Particles (65152105050250 and 4515210505250), mixed at a 1:1 ratio, were added to each sample. Then, 100% ethanol was added to each sample to achieve a final ethanol concentration of at least 50%. The samples were incubated with gentle shaking for 15 min and washed three times with 80% ethanol. Protein was eluted from SP3 beads using 200 mM EPPS (pH 8.5) containing Lys-C (Wako Chemicals, Richmond, VA, USA; Wako #129-02541). The samples were digested overnight at room temperature with vigorous shaking. The next morning, trypsin was added to each sample and further incubated for 6 h at 37 °C. Acetonitrile was added to each sample to achieve a final concentration of ~33%. Each sample was labeled with ~62.5 µg of TMTPro reagents (ThermoFisher Scientific, Waltham, MA, USA) in the presence of SP3 beads. Following the confirmation of satisfactory labeling (>97%), excess TMT was quenched by the addition of hydroxylamine to a final concentration of 0.3%. The full volume of each sample was pooled, and the acetonitrile was removed by vacuum centrifugation. The samples were acidified with formic acid and de-salted by StageTip eluted into autosampler inserts (ThermoFisher Scientific, Waltham, MA, USA), dried in a speedvac, and reconstituted with 5% acetonitrile and 5% formic acid for LC-MS/MS analysis.

Co-IP Liquid Chromatography and Tandem Mass Spectrometry—Mass spectrometry data were collected on an Orbitrap Eclipse mass spectrometer coupled to a Proxeon NanoLC-1000 UHPLC (ThermoFisher Scientific, Waltham, MA, USA). The 100 µm capillary column was packed in-house with 35 cm of Accucore 150 resin (2.6 μm, 150 Å; ThermoFisher Scientific, Waltham, MA, USA). Data were acquired for 180 min per run. A FAIMS device was enabled during data collection, and compensation voltages were set to −40 V, −60 V, and −80 V [74]. MS1 scans were collected in the Orbitrap (resolution—60,000; scan range—400–1600 Th; automatic gain control (AGC)—standard; and maximum ion injection time—automatic). MS2 scans were collected in the Orbitrap following higher-energy collision dissociation (HCD) (resolution—50,000; AGC—250%; normalized collision energy—36; isolation window—0.5 Th; and maximum ion injection time—100 ms).

Co-IP Mass Spectrometry Data Analysis—Database searching included all entries from the rat UniProt Database (downloaded in June 2022). The database was concatenated with one composed of all protein sequences for that database in the reverse order [75]. Raw files were converted to mzXML, and monoisotopic peaks were re-assigned using Monocle [76]. Searches were performed with Comet [77] using a 50 ppm precursor ion tolerance and fragment bin tolerance of 0.02. TMTPro labels on lysine residues and peptide N-termini (+304.207 Da), as well as carbamidomethylation of cysteine residues (+57.021 Da), were set as static modifications, while the oxidation of methionine residues (+15.995 Da) was set as a variable modification. Peptide-spectrum matches (PSMs) were adjusted to a 1% false discovery rate (FDR) using a linear discriminant, after which proteins were assembled further to a final protein-level FDR of 1% [78]. TMT reporter ion intensities were measured using a 0.003 Da window around the theoretical *m*/*z* for each reporter ion. Proteins were quantified by summing reporter ion counts across all matching PSMs. More specifically, reporter ion intensities were adjusted to correct for the isotopic impurities of the different TMTPro reagents according to the manufacturer’s specifications. Peptides were filtered to exclude those with a summed signal-to-noise (SN) value < 180 across all TMT channels and <0.5 precursor isolation specificity. The signal-to-noise (S/N) measurements of peptides assigned to each protein were summed (for a given protein). Data analysis based on the CRYAB Co-IP raw spectrum file can be found in the MassIVE database with the accession number MSV000097115.

Global Sample Preparation for Mass Spectrometry—CRYAB mouse heart samples for protein analysis were prepared as previously described [79,80]. Proteomes were extracted using a buffer containing 200 mM EPPS (pH 8.5), 8 M urea, 0.1% SDS, and protease inhibitors. Following lysis, 150 µg of each proteome was reduced with 5 mM TCEP. Cysteine residues were alkylated using 10 mM iodoacetamide for 20 min at RT in the dark. Excess iodoacetamide was quenched with 10 mM DTT. A buffer exchange was carried out using a modified SP3 protocol [73]. Briefly, ~1500 µg of Cytiva SpeedBead Magnetic Carboxylate Modified Particles (65152105050250 and 4515210505250), mixed at a 1:1 ratio, were added to each sample. Then, 100% ethanol was added to each sample to achieve a final ethanol concentration of at least 50%. The samples were incubated with gentle shaking for 15 min and washed three times with 80% ethanol. Protein was eluted from SP3 beads using 200 mM EPPS (pH 8.5) containing Lys-C (Wako, 129-02541). The samples were digested overnight at room temperature with vigorous shaking. The next morning, trypsin (ThermoFisher Scientific) was added to each sample and further incubated for 6 h at 37 °C. Following digestion, an equal volume of each sample was pooled to generate a pooled sample to be used as a bridge between two TMTPro 16-plex experiments. Acetonitrile was added to each sample to achieve a final concentration of ~33%. Each sample was labeled with ~300 µg of TMTPro reagents (ThermoFisher Scientific, Waltham, MA, USA) in the presence of SP3 beads. Following the confirmation of satisfactory labeling (>97%), excess TMT was quenched by the addition of hydroxylamine to a final concentration of 0.3%. The full volume of each sample was pooled, and the acetonitrile was removed by vacuum centrifugation for 1 h. The pooled sample was acidified, and peptides were de-salted using a Sep-Pak 50 mg tC18 cartridge (Waters, Milford, MA, USA). Peptides were eluted in 70% acetonitrile and 1% formic acid and dried by vacuum centrifugation.

Global Sample Phosphopeptide Enrichment—A phosphopeptide enrichment was performed using a High-Select Fe-NTA Phosphopeptide Enrichment Kit (ThermoFisher Scientific, Waltham, MA, USA). Dried phosphopeptides were de-salted using StageTips and re-dissolved in 5% formic acid/5% acetonitrile for LC-MS/MS. The flow-through from the phosphopeptide enrichment was used for total proteome profiling.

Global Sample Basic pH Reversed-phase Separation (BPRP)—TMT-labeled peptides were solubilized in 5% acetonitrile/10 mM ammonium bicarbonate (pH 8.0), and ~300 µg of TMT-labeled peptides were separated by an Agilent 300 Extend C18 column (3.5 μm particles: 4.6 mm ID and 250 mm in length). An Agilent 1260 binary pump coupled with a photodiode array (PDA) detector (ThermoFisher Scientific, Waltham, MA, USA) was used to separate the peptides. A 45 min linear gradient from 10% to 40% acetonitrile in 10 mM ammonium bicarbonate (pH 8.0) (flow rate of 0.6 mL/min) separated the peptide mixtures into a total of 96 fractions (36 s), which were consolidated into 24 samples in a checkerboard fashion and vacuum-dried to completion. Each sample was de-salted via StageTips and re-dissolved in 5% formic acid/5% acetonitrile for LC-MS3 analysis.

Global Sample Liquid Chromatography Separation and Tandem Mass Spectrometry (LC-MS2) for Proteomics—Proteome data were collected on an Orbitrap Fusion Lumos or Orbitrap Eclipse mass spectrometer (ThermoFisher Scientific, Waltham, MA, USA) coupled to a Proxeon EASY-nLC 1000 LC pump (ThermoFisher Scientific, Waltham, MA, USA). Fractionated peptides were separated using a 120 min gradient at 525–650 nL/min on a 35 cm column (i.d. of 100 μm, Accucore of 2.6 μm, and 150 Å) packed in-house. MS1 data were collected in the Orbitrap (ThermoFisher Scientific, Waltham, MA, USA; Fusion Lumos: 120,000 resolution, maximum injection time—50 ms, and AGC—6 × 10^5^; Eclipse: 60,000 resolution, maximum injection time—50 ms, and AGC—4 × 10^5^). Charge states between 2 and 6 (Fusion Lumos) or 2 and 5 (Eclipse) were required for MS2 analysis, and a 120 s dynamic exclusion window was used. The top 10 MS2 scans were performed in the ion trap with CID fragmentation (Fusion Lumos: isolation window 0.5 Da, Rapid, NCE—35%, maximum injection time—50 ms, and AGC—1.5 × 10^4^; Eclipse: isolation window 0.5 Da, Turbo, NCE—35%, maximum injection time—50 ms, and AGC—1 × 10^4^). For data collected on the Fusion Lumos, an online real-time search algorithm (Orbiter) was used to trigger MS3 scans for quantification [81]. MS3 scans were collected in the Orbitrap using a resolution of 50,000, NCE of 55%, maximum injection time of 200 ms, and AGC of 3.0 × 10^5^. The closeout was set to two peptides per protein per fraction [81]. For data collected on the Eclipse, a real-time search was used to trigger MS3 scans for quantification [81]. MS3 scans were collected in the Orbitrap using a resolution of 50,000, NCE of 55%, maximum injection time of 250 ms, and AGC of 1.25 × 10^5^. The closeout was set to two peptides per protein per fraction [81].

Global Sample Liquid Chromatography Separation and Tandem Mass Spectrometry (LC-MS2) for Phosphoproteomics—Phosphorylation data were collected on an Orbitrap Eclipse mass spectrometer (ThermoFisher Scientific) coupled to a Proxeon EASY-nLC 1000 LC pump (ThermoFisher Scientific). Fractionated peptides were separated using a 120 min gradient at 525 nL/min on a 35 cm column (i.d. of 100 μm, Accucore of 2.6 μm, and 150 Å) packed in-house. A FAIMS device was enabled during data acquisition, with compensation voltages set to −40, −60, and −80 V for the first shot and −45 and −65 V for the second shot [74]. MS1 data were collected in the Orbitrap (120,000 resolution; maximum injection time set to auto; and AGC of 4 × 10^5^). Charge states between 2 and 5 were required for MS2 analysis, and a 120 s dynamic exclusion window was used. The cycle time was set to 1 s. MS2 scans were performed in the Orbitrap with HCD fragmentation (isolation window 0.5 Da; 50,000 resolution; NCE of 36%; maximum injection time of 300 ms; and AGC of 2 × 10^5^).

Global Sample Data Analysis—Raw files were converted to mzXML, and monoisotopic peaks were re-assigned using Monocle [76]. Searches were performed using the Comet search algorithm against a mouse database downloaded from Uniprot in May 2021. We used a 50 ppm precursor ion tolerance, 1.0005 fragment ion tolerance, and 0.4 fragment bin offset for MS2 scans collected in the ion trap, with 0.02 fragment ion tolerance and 0.00 fragment bin offset for MS2 scans collected in the Orbitrap. TMTPro on lysine residues and peptide N-termini (+304.2071 Da) and carbamidomethylation of cysteine residues (+57.0215 Da) were set as static modifications, while the oxidation of methionine residues (+15.9949 Da) was set as a variable modification. For phosphorylated peptide analysis, +79.9663 Da was set as a variable modification on serine, threonine, and tyrosine residues. Each run was filtered separately to 1% FDR on the peptide-spectrum match (PSM) level. Then, proteins were filtered to the target 1% FDR level across the entire combined dataset. Phosphorylation site localization was determined using the AScorePro algorithm [82]. For reporter ion quantification, a 0.003 Da window around the theoretical *m*/*z* of each reporter ion was scanned, and the most intense *m*/*z* was used. Reporter ion intensities were adjusted to correct for isotopic impurities of the different TMTPro reagents according to the manufacturer’s specifications. Peptides were filtered to include only those with a summed signal-to-noise (SN) value ≥ 170 or 180 across all TMT channels. An extra filter of an isolation specificity (“isolation purity”) of at least 0.5 in the MS1 isolation window was applied for the phosphorylated peptide analysis. For each protein or phosphorylation site, the filtered peptide TMTPro SN values were summed to generate protein or phosphorylation site quantification values. The signal-to-noise (S/N) measurements of peptides assigned to each protein were summed (for a given protein). These values were normalized so that the sum of the signal for all proteins in each channel was equivalent, thereby accounting for equal protein loading. The resulting normalization factors were used to normalize the phosphorylation sites, again accounting for equal protein loading. Data analysis was based on the CRYAB Non-TAC Proteome 1–12 and Phospho 1–2 raw spectrum files, and the CRYAB TAC Proteome 1–12 and Phospho 1–3 raw spectrum files can be found in the MassIVE database with the accession number MSV000097115.

Cardiomyocyte Isolation and IonOptix—Wild-type *Cryab* and *Cryab^R123W^* mouse hearts were extracted, and single ventricular cardiomyocytes were isolated using the O’Connell and Simpson modification method and apparatus as previously described [83,84,85,86]. Single cardiomyocyte calcium transient and contraction dynamics were captured and analyzed using IonOptix equipment and software as previously described [85].

Data and Statistical Analysis—All bar plots, volcano plots, GO term plots, and heat maps were prepared in R v4.4.0 [54] using the following packages: ggplot2, dplyr, reshape2, ggpubr, tidyverse, RColorBrewer, ggrepel, clusterProfiler, DOSE, org.Mm.eg.db, enrichplot, ggupset, and pheatmap [87,88,89,90,91,92,93,94,95,96,97,98,99]. Global proteomic and phosphoproteomic data were analyzed using QIAGEN Ingenuity Pathway Analysis software (IPA) [55]. All statistical measurements are shown as mean ± standard deviation. All *p*-values were calculated using Student’s *t*-test for comparisons between 2 groups and two-way ANOVA for multi-group comparisons.

## Figures and Tables

**Table 1:** Common upstream regulators between co-immunoprecipitation and global proteomics predicted to be involved in the changes in protein expression, based on our mass spectrometry data, for wild-type CRYAB compared to CRYAB^R123W^ with and without transverse aortic constriction.

Enzymes	Kinases	G Protein-Coupled Receptors	Phosphatases	Ion Channels
DDX3X DHX36	EEF2K EGFR	ADORA2A ADRB1	PPP3CA PTP4A1	KCNJ2 MCOLN1
FAAH	EIF2AK4	AGTR2		SCN1B
HRAS	FLT1	CX3CR1		
KRAS	INSR	GPR174		
POLG	IPMK	MC5R		
	MAP2K6			
	MAP3K12			
	MAPK14			
	MKNK1			
	MOS			
	PRKAG3			
	PRKCZ			
	PTK2			
	RAF1			
	RET			
	ROCK1			
	ROCK2 TGFBR2 TK1 TTN			

## Data Availability

All proteomic data have been deposited in MassIVE under accession number MSV000097115.
